# COVID-19 and the *Wuhan Diary* –how does the overseas Chinese community react to group criticism?

**DOI:** 10.1007/s11366-021-09775-y

**Published:** 2022-01-08

**Authors:** Mei Wang, Marc Oliver Rieger

**Affiliations:** 1 Otto Beisheim School of Managment, WHU, Vallendar, Germany; 2 Department IV, University of Trier, Trier, Germany

**Keywords:** Uncritical patriotism, Conspiracy theory, Group criticism, Motivated reasoning and beliefs, Collective narcissism

## Abstract

We conduct an online survey to explore how Chinese people living in Germany perceive and react to group criticism in the context of the debate on the *Wuhan Diary*, a chronicle about life during the lockdown in Wuhan. We find that the majority rating of the book is a lukewarm “neither like nor dislike.” Most participants are open to criticism in principle and do not agree that the book only spreads so-called “negative-energy”. However, many participants were skeptical about the objectivity of the book and concerned about its potential use by so-called anti-China forces, even though the degree of blind patriotism is relatively low in our sample. The factors influencing the book’s evaluation are intriguing: perceived Western sentiment, media exposure and uncritical patriotism all affect COVID-19-related conspiracy beliefs, which in turn lead to a more negative evaluation of the book. A cluster analysis reveals two groups which differ in terms of properties like blind patriotism, belief in certain conspiracies, and also demographic parameters. Our results shed light on identity politics, motivated beliefs, and collective narcissism.

## Introduction

On 25 January 2020, two days after the COVID-19 lockdown was declared in Wuhan, a well-known Chinese author, Fang Fang (方方), started to post a daily account of Wuhan on social media. The chronicle almost instantly attracted enormous attention, often with polarized views. When it was announced that Fang Fang’s ‘Quarantine Diaries’ had been translated and published in English [1] and German [2], the opinions of many Chinese netizens shifted dramatically towards hostility. Many condemned Fang Fang for “handing a sword to anti-China forces.” [3]

Although the diary occasionally raised some issues on the accountability of the local government and mentioned some daily troubles during the lockdown, it also praised the courage and resilience of people in Wuhan [4]. One may therefore wonder whether the hypersensitivity to criticism results from state propaganda and censorship. In this study, we approach the issue from a different angle, by looking at the Chinese community in Germany. Focusing on overseas Chinese helps us to circumvent the potential self-censorship problem with surveys in China, as we can expect that overseas Chinese are more likely to express their true opinions. Moreover, given that there are no restrictions to obtaining information from independent sources, Chinese people in Germany are fairly free to form their own opinions. This approach echoes Carlson’s call for scholarly attention to the role of “boundary-spanners”, referring to those who subscribe to plural identities [5], for example the Chinese Protestants studied by Entwistle [6]. Chinese communities in Western countries have a different media and cultural environment as compared to domestic Chinese people. This study could help us to gain more insight into the fundamental sociopsychological mechanisms involved in forming beliefs and attitudes, even in a different media and political environment.

Studies on Chinese nationalism and patriotism tend to focus on the role of contemporary political influence, historical origins, different types of patriotism, and the demography of patriots [7]. There is a lack of studies, however, on Chinese patriotism from a more fundamental sociopsychological perspective. Some studies show that Chinese patriots are on the one hand highly educated, rational and pro-democratic, but on the other hand xenophobic, extremely sensitive to criticism, and constantly demanding apologies for any alleged offense [8]. A cognitive-motivational framework of motivated reasoning may help us to reconcile such seemingly contradictory behavior [9]. Our study focuses on the interrelation of patriotism, beliefs, and attitudes in terms of group-directed criticisms. By surveying an overseas community, we seek to identify more general patterns of patriotism, independent of political regimes.

As suggested by Fukuyama, it is easier to understand current politics if we shift the focus from political ideologies to beliefs about identities, in particular national and ethnic identities [10]. Identity attachment helps to satisfy the basic human need for belonging to a bigger group. Group criticism can be perceived as constructive or destructive to the ingroup. Previous studies show that group criticism from insiders is better tolerated, but not when the group perceives itself as being under threat [11]. Blind patriotism, a strong form of uncritical attachment to one’s nation, “whether right or wrong,” tends to increase the perceived external threat, which is unrelated to the real threat [12–14]. In the case of the *Wuhan Diary*, although Fang Fang is a respectable insider, a majority of our respondents worried that the book could be used by “anti-China forces”.

The controversy surrounding the *Wuhan Diary* offers us an opportunity to explore an interesting question: in an open society, when one is exposed to more diverse information and value systems, to what extent is one open to different voices? In fact, most of our respondents claimed to be tolerant to criticism and believed that open discussion of shortcomings can improve a society. At the same time, however, our respondents who were more negative about the book also tended to believe that COVID-19 originated in the U.S. and perceived the book as not being objective. This suggests that one mechanism for reconciling open-society value and opposition to group criticism is through the formation of beliefs in favor of the ingroup and rejection of criticism as a fabrication. In other words, strong attachment to group identity (e.g., blind patriotism) can lead to hypersensitivity to group criticism through motivated beliefs. Although experimental studies find that reminders of the importance of free speech effectively increase openness to criticism [11], our results imply that blind patriots can endorse freedom of speech but simultaneously reject group-directed criticism as implausible and dubious. Such beliefs are more likely to be influenced by emotional attachment rather than factual reasons. Therefore, motivated beliefs can be important barriers to openness to dissenting voices, no matter whether these voices are from insiders or outsiders.

The remaining part of the article is structured as follows. We first connect the literature on social identity, group criticism, and motivated beliefs. We also form our hypotheses based on the insights from the literature. Afterwards, we present our survey methodology and the empirical results. Finally, we conclude and discuss the implications of our results.

## Social identity, group criticism, motivated beliefs: A literature review

Chinese nationalism has attracted enormous attention in recent decades. Most studies tend to portray Chinese nationalism as a special phenomenon that can be attributed to Chinese history and contemporary Chinese politics. Although insightful, this perspective isolates Chinese scholars from the broader discussion on nationalism and identity politics [5]. It also limits our understanding of the sociopsychological processes underlying Chinese nationalism which apply to all human beings. Taking a bottom-up approach, our study intends to bridge the gap by adopting a broader view based on sociopsychology literature to understand the potential impacts of national identity attachment on beliefs and attitudes.

It has been observed that Chinese people tend to be oversensitive to criticism of China, even if they are themselves critical of the current politics [15]. Using a survey related to the *Wuhan Diary*, we investigate the underlying drivers of the attitude towards group criticism. In particular, we focus on the role of beliefs and blind patriotism. Our study therefore bridges three strands of literature—social identity, group criticism, and motivated belief.

Social identity is a fundamental aspect of human existence. It refers to the self-concept derived from the perceived membership in a relevant social group [10, 16]. Past literature shows that identity and motivation of the critics are important predictors of the reaction to group criticism [12]. People tend to be more defensive to criticism from outgroup members (‘It’s ok if we say it, but you can’t.’) [17]. Studies find this intergroup sensitivity effect in the context of criticism directed towards nations [12, 18]. Typically, people are more open to insiders’ criticism, because their motivation is perceived as being more constructive [18]. People become more defensive, however, when they perceive external threats and when insiders’ criticism is given by outsiders. In these cases, the motivation of critics is more suspicious to ingroup members. For example, interviews with Chinese overseas students reveal that some of them tend to be uncomfortable with discussing the dark side of China with foreigners, even if they are critical about those issues themselves [15].

Consistent with the group criticism literature, one reaction to the *Wuhan Diary* relates to the author’s motivation. People often question the motivation of critics (‘You can criticise because you care.’) [12]. Fang Fang’s identity (insider) and motivation (helping to improve) were more positively perceived at the beginning. A noticeable turning point for many of her supporters was when the diary was translated into English and German. Even if the group is convinced of the critic’s honourable motives, the critic may still encounter resistance if they violate formal or informal “rules” [17]. According to Hornsey, two such rules are that “criticism should be kept ‘in-house’ and that people should not criticise their group when it is facing threat from the outside (e.g., in times of war).” [17] The translation of Fang Fang’s diary seems to violate both implicit rules: the criticism is brought outside China and this is generally unacceptable when people perceive the Western world as hostile to China. Some Chinese people even started to question Fang Fang’s initial motivation. This is consistent with past research which shows that people are less open to criticism when it is aired publicly to an outgroup audience [19].

Another typical reaction to the *Wuhan Diary* is to question its credibility. This is related to how people form their beliefs on accuracy of facts. If people updated beliefs rationally, then more information would lead to convergence of opinions. In reality, however, we often observe polarized views even in an environment with abundant information. Despite facing identical information, people can form different opinions. This can be attributed to *motivated reasoning and beliefs*, which refers to the psychological phenomenon that people choose to believe what they want to believe [3, 20, 21]. One notable example is the impact of identity attachment on belief formation. With a national survey of Americans, Herrmann finds that stronger attachment to the nation can lead to polarized political beliefs on issues like globalization and Middle East politics [22]. By rewriting reality, people manage their beliefs to make them logically consistent and compatible with their own preferences. Similarly, belief in intergroup conspiracies can be motivated by irrational and emotional attachment to group identities [23]. For example, results from two national surveys in the U.S. and the U.K. suggest that national narcissism—a tendency to exaggerate the greatness of one’s nation— increased the proneness to believe and disseminate conspiracy theories during the COVID-19 pandemic [24].

Based on the literature on social identity, group criticism and motivated beliefs, we designed our study to measure these aspects. We measure a special type of social identity attachment, *blind patriotism*, i.e., an uncritical attachment to the nation. We also explore how blind patriotism relates to a set of beliefs that are frequently circulated in Chinese social media concerning COVID-19 and the *Wuhan Diary*: (1) intergroup conspiracies (“*the virus is from the U.S./it was brought to Wuhan by CIA*”); (2) credibility of the critics (“*the book is not objective*”); (3) perceived foreign threat (“*the book can be used by anti-China forces*”); (4) perceived negative sentiment of the book (“*the book has too much negative energy*”).^1^ Finally, we elicit a general assessment of the book. We expect social identity, as measured by blind patriotism, to affect beliefs and perceptions of the book, which in turn predict the general assessment of the book. More specifically, we hypothesize that (1) blind patriotism increases the tendency towards intergroup conspiracy beliefs; (2) intergroup conspiracy beliefs predict a more negative perception of the book, i.e., lower credibility, higher potential foreign threat, higher “negative energy”.

## Data and methodology

### Survey procedure

We conducted an online survey among the Chinese community in Germany. Targets were first- and second-generation Chinese immigrants. The survey language was Chinese, thus excluding second-generation immigrants who were not fluent in reading Chinese. One reason is that the intense discussion about the *Wuhan Diary* had been mostly in Chinese language. The Chinese community in Germany does not have any central organizational structure that could have been used to advertise the survey, and thus we relied on distributing the link via WeChat and email listings with the help of colleagues and Confucius Institutes. The survey was conducted in two waves, in May and August 2020, where the composition of survey items was slightly adjusted. This survey is part of a larger survey project [27]. This project included a number of additional questions, mainly about the situation of Chinese people in Germany during COVID-19, which will be pursued in other research. As incentive to participate, five €20 Amazon coupons were given to randomly selected participants. In total, 193 participants filled in the survey (91 in May and 102 in August), out of which 142 completed the main part. The participants of the main survey were asked at the end whether they would be willing to also answer a few questions about the *Wuhan Diary*, and 112 agreed to answer. In the second wave, we asked all participants whether they had ever heard of the book, and 74% said they had.

### Questionnaire

In this section, we provide the wordings of the most relevant items for our research (translated into English) and the definitions of survey variables. We skip simple demographic questions. Codebooks with the wordings of all items in Chinese language can be provided upon request.

We asked the two questions related to intergroup conspiracies about the origin of COVID-19. We also elicited level of agreement with various statements related to blind criticism, intergroup conspiracies, assessment of the book, and other aspects.

Intergroup conspiracies:

*From where do you think COVID-19 originates?* (No / Rather unlikely / Rather likely / Yes).

*China*

*USA*

*Elsewhere*

*In your opinion, how likely is the following theory about COVID-19?*

*The U.S. Secret Services developed the virus and imported it into Wuhan to damage China.* (Very unlikely / Unlikely / Somewhat likely / Likely / Very likely).

Blind patriotism:

We should all fight for our country (China) whether it is right or wrong.

(Totally disagree / Somewhat disagree / Somewhat agree/ Totally agree).

Tolerance of criticism:

I am not in favor of discussing the dark side of society in the public sphere.

Without openly discussing negative issues, there is no way to improve the situation.

(Totally disagree / Somewhat disagree / Somewhat agree/ Totally agree).

Assessment of the diary:

*This diary only spreads negative energy.*

*The diary is quite objective.*

*The diary can be used by anti-China forces.*

(Totally disagree / Somewhat disagree / Somewhat agree/ Totally agree).

*What is your overall evaluation of the diary?*

(Very negative / Negative / Neutral /Positive / Very Positive).

Shame and blame:

*If COVID-19 really originated in *China*, I would feel very ashamed.*

*No country should be blamed because of the origin of a pandemic.*

(Totally disagree / Somewhat disagree / Somewhat agree/ Totally agree).

Perceived Western sentiment towards China^2^:

*Western society is happy to see *China* decaying.*

*Most Westerners understand the feelings of Chinese people.*

(Totally disagree / Somewhat disagree / Somewhat agree/ Totally agree).

Media consumption:

*What are your main sources of media for news?*

(WeChat / Chinese TV or radio / Chinese newspaper or internet media / Facebook / German TV or radio / German newspaper or internet media / TV or radio in English or other European language / Newspaper or internet media in English or other European language).

We computed the difference between the number of selected Chinese language news media and the number of selected non-Chinese language news media and denoted the resulting variable as “Chinese vs. Western media”.

Aside from the number of years that subjects lived in Germany, we also elicited information on the following items about integration:

*In Germany, do you have more Chinese friends or more German friends?*

*(More Chinese friends / About the same / More German friends)*

*What is your German level? (A1/A2/B1/B2/C1/C2)*

## Results

### Descriptive statistics on various measures of attitudes and behavior

#### Reading experience and evaluation of the diary

More than half (52.5%) of all respondents have read the diary completely or partially. The average evaluation of the book is 2.9 on a 5-point Likert scale (1 = dislike very much; 5 = like very much). In total, 25% of participants stated that they disliked the book vs. 17% who liked it, whereas more than half (58%) of our respondents chose the rather neutral rating “neither like nor dislike” (= 3). It is interesting to see that among the participants who have *not* read the diary, around 28% disliked the book, but only 2% liked it. Among the people who have read the diary “a bit”, the proportion of like vs. dislike seems to be more balanced (24% like it vs. 20% dislike it). A small proportion of our participants (14%) have read most or all of the diary, and their overall rating of the book tends to be higher, but these two subgroups (“read most” or “read all”) are too small to make any conclusive comparisons with other groups. See Table 1 for more details.

**Table 1:** Summary of reading experience and overall evaluation of the diary

	not read	read a bit	read most	read all	Total	Customer reviews (Dec 20, 2020)	Amazon.de (German)	Amazon.com (English)
1 = dislike very much	11.8%	6.5%	16.7%	0.0%	9.8%	1 star	9%	31%
2 = rather dislike	15.7%	13.0%	16.7%	33.3%	15.2%	2 star	7%	3%
3 = neither like nor dislike	70.6%	56.5%	25.0%	0.0%	58.0%	3 star	8%	6%
4 = rather like	2.0%	21.7%	8.3%	0.0%	10.7%	4 star	16%	7%
5 = like very much	0.0%	2.2%	33.3%	66.7%	6.3%	5 star	60%	53%
Mean	2.6	3.0	3.3	4.0	2.9		4.1	3.5
Std. Dev	0.7	0.8	1.5	1.7	1.0			
N	51	46	12	3	112		110	234
Proportion	47.5%	39.0%	10.2%	3.4%	100%			

As an interesting comparison, we also present the customers’ ratings (up to Dec 20, 2020) of the German translation on amazon.de and the English translation on amazon.com in the last two columns of Table 1. The German version received a (weighted) average rating of 4.1, with 60% 5-stars (best), and only 9% 1-star (worst). The English version on amazon.com, however, seemed to attract bipolar evaluations, with 53% 5-stars vs. 30% 1-star, leading to a substantially lower rating of 3.5 out of 5. We observed some notable differences between the negative (1-star) comments on amazon.com and amazon.de: the former are more often written by Chinese readers (as can be seen from the names), tend to be more emotional, and are dominated by the three types of arguments we focused on in our survey (i.e., objectivity, anti-China force, and negative energy). In comparison, the negative comments on the German version are more often by non-Chinese readers with much more diverse opinions, from “boring” to “too pro-government.” The striking difference in the proportions of worst rating (31% English vs. 9% German) seemed to be driven by similar statements from English-speaking Chinese people. Prototypical examples are comments like “the book is based on hearsay” or “all lies”, which are critiques often circulated in Chinese domestic social media. This is further supported by the observation that on the French webpage amazon.fr and the Spanish website amazon.es, the average ratings for the various translations are also all substantially better than on amazon.com. As a further comparison, we also checked an influential and popular novel depicting Chinese modern political history, *Wild Swans: Three Daughters of China*, and did not find such a dramatic difference in amazon ratings of the English and German versions—both versions were rated 4.6 out 5. The most likely reason for this contrast is that this novel first appeared in English and has been completely censored in mainland China. Hence, the book remains unknown to most Chinese people despite its popularity outside China.

In total, 68% (± 4%) of our respondents disagree with the statement that “the diary only spreads negative energy”, but 62% (± 4%) did agree that the book “can be used by anti-China forces”. Regarding how objective the diary is, less than half (40.5% ± 4%) of participants believed that it is at least relatively objective. In summary, the majority belief is that the book is not dark, but it lacks objectivity and can be used by anti-China forces. See Table 2 for more details.

**Table 2:** Assessment of the diary and other issues

	Mean	Std. Dev	totally disagree (= 1)	rather disagree (= 2)	rather agree (= 3)	totally agree (= 4)
*Assessment of the diary*						
This diary only spreads negative energy	2.14	0.93	27.4%	40.7%	22.1%	9.7%
This diary is relatively objective	2.30	0.87	18.9%	40.5%	32.4%	8.1%
This diary will be used by anti-China forces	2.72	0.99	14.3%	23.2%	38.4%	24.1%
*Blind patriotism*						
We should all fight for our country (China) whether it is right or wrong	2.04	0.94	34.3%	35.7%	22.4%	7.7%
*Perceived Western sentiment towards China*						
Western society is happy to see China decaying	2.42	0.79	11.7%	42.0%	38.9%	7.4%
Most Westerners understand the feelings of Chinese people	2.20	0.68	14.2%	51.9%	33.3%	0.6%
*Open discussion of negative issues*						
I am not in favor of discussing the dark side of society in the public sphere	1.99	0.79	29.4%	44.1%	24.5%	2.1%
Without openly discussing negative issues, there is no way to improve the situation	3.01	0.78	2.1%	23.1%	46.2%	28.7%
*Shame and blame*						
If COVID-19 really originated in China, I would feel very ashamed	1.60	0.78	55.6%	31.5%	10.5%	2.5%
No country should be blamed because of the origin of a pandemic	3.64	0.67	1.9%	5.6%	19.1%	73.5%

#### Blind patriotism and perceived Western attitudes towards China

Table 2 shows that about 70% (± 4%) of our participants disagree with the blind patriotism statement “We should all fight for our country (China) whether it is right or wrong.” More than half (54% ± 4%) of the respondents disagree with the statement “Western society is happy to see China decaying.” However, two-thirds of the participants (66% ± 4%) also disagree with the statement “Most Westerners understand the feelings of Chinese people.”

#### Attitudes towards public discussion of negative issues

Table 2 also shows that our respondents claimed to be relatively open to the public discussion of negative issues. In total, 63% (± 4%) of our participants disagreed with the statement “I am not in favor of discussing the dark side of society in the public sphere.” and 75% (± 3%) agreed that “without openly discussing negative issues, there is no way to improve the situation.”

#### Blame and Shame

Our participants also have relatively high consensus regarding the shame and blame related to the pandemic. A dominant majority (87% ± 3%) disagreed with the statement that “if COVID-19 really originated in China, I would feel very ashamed.”, and nearly all (93% ± 2%) agreed that “no country should be blamed because of the origin of a pandemic.” See the last two lines in Table 2.

#### Usage of media and beliefs about COVID-19 origin

Table 3 shows that WeChat is the most widely used media as a news source (77% ± 3%), followed by the Chinese press (67% ± 4%) and the German press (60% ± 4%). Regarding the location of origin of COVID-19, 49% (± 4%) believed it is likely or definitely from China, and 58% (± 4%) believed it is likely or definitely from the United States. The right column in Table 3 shows the perceived likelihood of the conspiracy story that the virus was designed and distributed by the CIA. Although a majority of our participants (63% ± 4%) stated that this is almost or rather impossible, 23% (± 3%) chose a moderate possibility, 12% (± 3%) believed it is rather likely, and 1.4% (± 1%) thought it is highly possible.

**Table 3:** Descriptive statistics on media consumption and beliefs about origins of COVID-19

Using these media as news source:			Belief about origin of COVID-19:		Belief in the conspiracy theory that CIA designed and distributed the virus	
WeChat	77%		*Origin in China*			
Chinese press	67%		definitely not	14.2%	almost impossible	31.0%
German press	60%		unlikely	36.5%	rather unlikely	31.7%
German TV	50%		likely	43.9%	moderate possibility	23.4%
Chinese TV	35%		yes, definitely	5.4%	rather likely	12.4%
English press	30%		*Origin in the US*		highly possible	1.4%
English TV	28%		definitely not	4.1%		
Facebook	21%		unlikely	37.4%		
			likely	50.3%		
			yes, definitely	8.2%		

### Correlation analysis

Table 4 exhibits the correlation structure of the various measures. As we expected, participants with a higher degree of blind patriotism tend to believe that COVID-19 more likely originated in the U.S. and was designed by the CIA. They are also less likely to have read the diary, and have more negative opinions about the book.

**Table 4:** Correlation matrix of beliefs and attitudes

	Blind patriotism	Perceived positive Western sentiment	Follow news on Chinese vs. Western media	Belief in origin in U.S. vs. China	Belief in CIA conspiracy	Read the book	Book: “negative energy”	Book: objective	Book: “anti-China forces”
Perceived positive Western sentiment	-0.13								
Follow news on Chinese vs. Western media	0.12	-0.001							
Belief in origin of COVID-19 in U.S. vs. China	0.36**	-0.30**	0.26**						
Belief in CIA conspiracy	0.33**	-0.34**	0.21*	0.55**					
Read the book	-0.20*	0.09	0.09	-0.11	-0.07				
Book: “negative energy”	0.41**	-0.40**	0.27**	0.44**	0.51**	-0.11			
Book: objective	-0.39**	0.36**	-0.04	-0.42**	-0.34**	0.33**	-0.52**		
Book: “anti-China forces”	0.35**	-0.43**	0.13	0.50**	0.44**	-0.16	0.60**	-0.59**	
Book: overall evaluation	-0.39**	0.33**	-0.12	-0.56**	-0.45**	0.30**	-0.58**	0.70**	-0.63**

***, **, * represents significance at 0.1%, 1%, 5% level, respectively. Standard error in the parentheses.

Table 4 also suggests that the beliefs about the origin of COVID-19 and the CIA conspiracy story correlate with perceived Western sentiment towards Chinese as well as the assessment of the book. The participants who follow news more in Chinese media than in Western media are also more likely to believe that COVID-19 originated in the U.S. and that it was made by the CIA.^3^ We now take a closer look at the predictors of opinions regarding the diary as well as beliefs about the origin of COVID-19 by using regression analysis.

### Regression analysis

#### Blind patriotism and intergroup conspiracy belief

As we expected, blind patriotism is significantly correlated with proneness to believe in intergroup conspiracies (i.e., COVID-19 is more likely to have originated in the U.S.), as shown in the first column of Tables 5 and 6. This is consistent with the previous literature on social identity and beliefs [24]. The proneness to intergroup conspiracy beliefs is also associated with more Chinese media consumption, fewer German friends, and more perceived negative Western sentiment. Blind patriotism is nevertheless significant after controlling for these factors.

**Table 5:** Regression analysis on evaluation of diary and beliefs about origin of COVID-19

	Origin U.S. vs. China	Objective	Anti-China forces	Negative energy	Overall evaluation of Wuhan diary
Belief in origin of COVID-19 in U.S. vs. China		-0.28** (0.07)	0.36*** (0.08)	0.23* (0.08)	-0.31*** (0.07)
Objective					0.37*** (0.10)
Anti-China forces					-0.14 (0.09)
Negative energy					-0.18 (0.10)
Blind patriotism	0.33*** (0.10)	-0.26** (0.09)	0.16 (0.10)	0.22* (0.10)	0.00 (0.09)
Follow news on Chinese vs. Western media	0.23** (0.29)	0.01 (0.24)	-0.03 (0.27)	0.25** (0.26)	0.06 (0.22)
Perceived positive Western sentiment	-0.31*** (0.07)	0.18 (0.07)	-0.32** (0.07)	-0.25** (0.07)	-0.04 (0.06)
August survey wave	0.05 (0.19)	0.05 (0.17)	-0.05 (0.19)	0.15 (0.18)	0.12 (0.15)
Years living in Germany	-0.11 (0.02)	0.21* (0.02)	-0.1 (0.02)	0.06 (0.02)	0.11 (0.02)
German level	0.11 (0.15)	-0.03 (0.12)	-0.11 (0.14)	-0.05 (0.13)	0.05 (0.11)
More German friends	-0.16* (0.14)	-0.22* (0.12)	0.17 (0.13)	0.22* (0.12)	-0.07 (0.10)
Demographic controls	Yes	Yes	Yes	Yes	Yes
R^2^	0.34	0.40	0.43	0.43	0.60
N	131	102	102	103	102

***, **, * represents significance at 0.1%, 1%, 5% level, respectively. Standard error in the parentheses

**Table 6:** Regression analysis on overall evaluation of Wuhan diary

	Overall evaluation of Wuhan diary		
	Model 1	Model 2	Model 3
Belief in origin of COVID-19 in U.S. vs. China	-0.24** (0.06)	-0.25** (0.06)	-0.31*** (0.07)
Book: objective	0.38*** (0.09)	0.39*** (0.10)	0.37*** (0.10)
Book: can be used by anti-China forces	-0.18* (0.09)	-0.20* (0.09)	-0.14 (0.09)
Book: “negative energy”	-0.15 (0.09)	-0.16 (0.09)	-0.18 (0.10)
Read the book	0.11 (0.08)	0.11 (0.08)	0.13 (0.09)
Perceive that Germans are positive about Chinese		-0.06 (0.06)	-0.04 (0.06)
Follow news on Chinese vs. Western media		0.01 (0.19)	0.06 (0.22)
Blind patriotism		0.01 (0.08)	0.00 (0.09)
August survey wave			0.12 (0.15)
Years living in Germany			0.11 (0.02)
German language level			0.05 (0.11)
More German friends			-0.07 (0.1)
Age			-0.17 (0.01)
Female			0.02 (0.15)
Student			-0.08 (0.2)
University degree			0.09 (0.23)
Working			-0.03 (0.18)
adjusted R^2^	0.61	0.60	0.60
N	102	102	102

***, **, * represents significance at 0.1%, 1%, 5% level, respectively. Standard error in the parentheses.

#### Intergroup conspiracy belief and judgment of group criticism

How is conspiracy belief related to judgment about the book? This can be seen as second-order motivated reasoning and beliefs. Table 5 shows that conspiracy believers tend to perceive the book as less credible, more vulnerable to foreign threats, and emitting only “negative energy”, which is consistent with our hypothesis.

It is not surprising to see that participants who follow more Chinese media are more likely to agree that the book spreads only “negative energy”, a catchphrase which has been hijacked by Chinese leadership to enhance legitimacy and discourage criticism in the last decade [19].

#### Beliefs and overall evaluation of the book

The last column in Table 5 shows that conspiracy belief is a significant predictor of overall evaluation of the book, i.e., people who believe in the U.S. origin of COVID-19 are more likely to hold a lower opinion of the diary.

Although Table 4 shows that all three aspects of judgment of the book (objectivity, anti-China force, and negative energy) are significantly related to overall evaluation of the book, when all three variables entered the regression, objectivity is the most robust predictor of the overall evaluation of the book. The lack of statistical significance of the other two aspects (anti-China force and negative energy) is probably due to the relatively small sample size, but the sign of these variables is as expected.

All other factors such as perceived Western sentiment, news source, and other demographic variables play a less important role in predicting the overall evaluation of the diary.

### Cluster analysis

In order to better depict the sociodemographic profile of our participants, we also conducted a cluster analysis to categorize our respondents based on the following variables: blind patriotism, beliefs about the origin of COVID-19 and in the CIA conspiracy, use of Chinese vs. Western media, perceived positive Western sentiment, years living in Germany, and German level.

The analysis reveals two clusters with 43% vs. 57% in each cluster respectively (Table 7). Although most of our participants are relatively young and highly educated, the two clusters have distinctive features in many aspects: The first group disagrees more with blind patriotism and is more open to discussions of the dark side of China. The first group has lived in Germany much longer than the second group (7.4 vs. 3.7 years) and perceives Western sentiment towards China as more positive, whereas the second group relies more on Chinese media (0.33 vs. 0.16) and is more prone to intergroup conspiracy beliefs.

**Table 7:** Cluster analysis

	Average values		T-test
Clustered variables	Cluster 1	Cluster 2	Significance
N	57	76	
%	42.9%	57.1%	
Blind patriotism	1.68	2.34	***
U.S. vs. Chinese origin	-0.56	0.80	***
CIA conspiracy	1.54	2.71	***
Chinese vs. Western media	0.16	0.33	***
Perceived positive Western sentiment	0.28	-0.71	***
Years living in Germany	7.44	3.66	***
German level	2.32	2.13	
Other variables			
Book: “negative energy”	1.78	2.47	***
Book: objective	2.61	2.07	***
Book: can be used by anti-China forces	2.28	3.07	***
Overall evaluation on the diary	3.22	2.59	***
I am not in favor of discussing the dark side of society in the public sphere	1.65	2.24	***
Without openly discussing negative issues, there is no way to improve the situation	3.19	2.92	*
If COVID-19 really originated in China, I would feel very ashamed	1.56	1.67	
No country should be blamed because of the origin of a pandemic	3.72	3.63	
age	35.5	29.8	***
female	75%	58%	*
student	25%	54%	***
University degree	91%	87%	

* = 5% level, ** = 1% level, *** = 0.1% level

significantly larger values in bold face

The difference in media consumption means that the first group marked on average 0.33 more non-Chinese media types used for news coverage than Chinese-media types, the second group only 0.16. This difference, although statistically significant, does not seem as large as one would a priori expect. We did not, however, elicit the amount and type of news that respondents read from the various sources. It seems plausible that the difference would be more visible when measured more accurately.

Regarding the difference in the time spent in Germany between the two groups, it is of course unclear whether this effect is based on different cohorts (the younger generation in China being more patriotic) or an effect of longer exposure to the German surrounding.

Given the lower degree of belief in intergroup conspiracy beliefs and their lower uncritical patriotism, it is not a surprise that the first group rated the book as more objective, disagreed more with the “negative energy” statement, and was less concerned about potential threats from “anti-China forces.” The overall evaluation of the book from the first group is significantly more positive than that of the second group.

### Overview of results

**Fig. 1:**
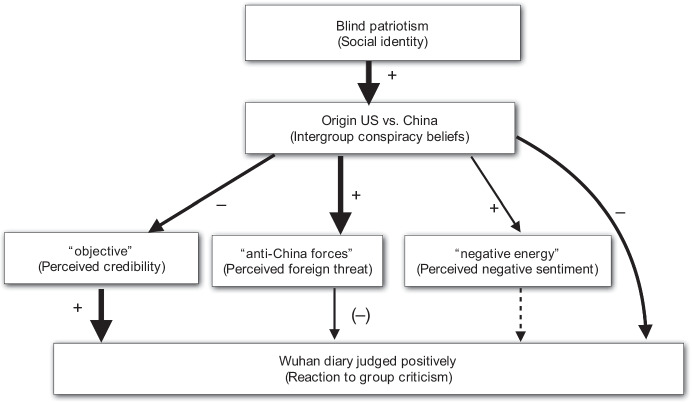
Overview of general results: arrows mark potential causal effects where the width of the lines marks the size of the measured relations. Dashed line represents a non-significant relationship

The main hypotheses and results are summarized in Fig. 1, where the strength of the arrows reflects the strength of the effects. Consistent with our expectations, we find that blind patriotism, an uncritical attachment to one’s nation, is positively associated with intergroup conspiracy beliefs. We consider the beliefs about different aspects of the book as secondary beliefs based on identity-oriented intergroup beliefs. As expected, intergroup conspiracy beliefs not only directly predict the overall evaluation of the book, but also predict the perception of the book in terms of its credibility, vulnerability to foreign threat, and alleged “negative energy”, of which only perceived credibility is a robust predictor for the overall evaluation of the book.

We also find a slight “separation” of two prominent clusters when looking at various demographic parameters, uncritical patriotism and intergroup conspiracy beliefs: One group was less prone to intergroup conspiracy beliefs and uncritical patriotism. This group consisted on average of more women and older people who had already stayed for a longer period in Germany. The second group, accordingly, had the opposite characteristics.

To summarize, our findings suggest that strong and blind attachment to social identity is important in shaping belief in intergroup conspiracies and suspicion towards the credibility of critics, leading to resistance to group criticism (‘We are good, and we don’t believe you if you disagree’).

## Conclusion and Discussion

The rise of nationalism on a global scale during COVID-19 has become a big concern [8, 20, 21, 28–34]. While the nationalist sentiment reflected in the debate about the *Wuhan Diary* has been most intense in China, it sent ripples through Chinese communities worldwide. Around three quarters of our Chinese respondents in Germany had heard about the book. This highlights the fact that discussions about the book were clearly not restricted to literate circles, but rather were ubiquitous. Our survey did not show a strong bipolar division regarding the overall evaluation of the *Wuhan Diary*. Most of our participants expressed rather neutral or lukewarm opinions about the book. It is not clear whether this ‘silent majority’ reflects true indifference or whether they would like to distance themselves from the ongoing arguments.

It is not our intention to take sides on the debate around the *Wuhan Diary*, but to use this opportunity to better understand the potential deeper sociopsychological reasons behind the debate. Diaspora Chinese belong to the so-called “boundary-spanners” due to their plural social identities [5]. Studying this group helps us to enrich our understanding of the identity and belief formation behind patriotism and nationalism.

Even though nationalist sentiments seem to be on the rise in recent decades [7], only about 30% of our participants were blindly patriotic (“fight for my country whether right or wrong”). This is substantially lower than the 50% of a domestic Chinese sample reported in another recent study, although the comparison should be taken with caution as neither sample is representative [35]. Most of our respondents also expressed support for freedom of expression and openness to criticism. Consensus on these principles is crucial for building civic societies. Discourse analysis by Zhao reveals that Chinese domestic netizens have been “rational but xenophobic” during the pandemic [36]. This is in line with our findings that overseas Chinese are generally open to criticism, but at the same time are afraid of potential foreign threat. The popular official narratives promoted by the Chinese regime, such as that of “negative energy”, are not particularly appealing to our overseas participants. They are also not significant in predicting the overall rating of the book. Taken together, we agree with Liu’s observation from more than a decade ago that the revival of overseas Chinese nationalism is not necessarily leading to “a unified ideology or a movement with centralized leadership such as that in the 1930s” [7].

More exposure to Chinese media is associated with agreement that “the diary only spreads negative energy” as well as the tendency to believe that the virus originated in the United States. These media consumption habits and beliefs could be driven by identity attachment and reinforce each other —people choose the news which are in favor of their group, which reinforces their previous beliefs and emotional attachment. It is also worth mentioning that one obstacle to searching for information is that many Chinese people, domestic or overseas, are not aware of the sophisticated art of censorship. Very much to the contrary, they think that the hot debate surrounding Fang Fang shows exactly how open the media environment is in current China. If they only follow domestic Chinese media, they would not know that several citizen journalists (e.g., Fang Bin, Chen Qiushi, Zhang Zhan, and Li Zehua) have been arrested or have disappeared since early February 2020 after they made an effort to report independently on the situation in Wuhan. As Wu Qiang, an independent political analyst based in Beijing, commented on the *Wuhan Diary* in an interview, “Many voices from Wuhan have been silenced. The fact that her work was allowed to survive is the art of censorship: to let out a relatively moderate voice to avoid the embarrassment of a completely blank canvas.”[4] A recent large-scale survey on 8000 students from university students in Wuhan reveals high demand for transparency of government’s information disclosure [37]. It would be interesting for future studies to compare overseas and domestic Chinese concerning their attitudes towards information transparency.

The perceived objectivity of the book seems to have been influenced by a number of conspiracy theories that have been spreading on social media regarding the origin of COVID-19. Fang Fang, living next door to the initial outbreak, obviously describes Wuhan as the starting point of the pandemic. However, from March 2020 on, the “narrative battle” between China and the U.S. has shifted to disputes about the origin of the virus, generating various versions of conspiracy theories from both sides, such as the CIA or a Chinese bioweapon as origins [27]. As a result, at present, a majority of Chinese people seem to firmly believe that the pandemic did not start in China, but rather elsewhere. As documented in a representative survey conducted in China, about 53% of Chinese respondents believed that the coronavirus is a bioweapon developed by the United States and brought intentionally to Wuhan [38]. Similarly, a majority of our Chinese respondents in Germany suspect an American origin of COVID-19, whereas other studies show that more German respondents tend to believe in the story that the virus is part of a Chinese bioweapon program [39, 40], even though both stories lack scientific evidence. Our survey provides additional indirect evidence of power of the global narrative [27, 41]. Such blame narratives represent a barrier to mutual trust and the urgently needed global collaboration, especially in times of a global health crisis.

In addition, our results contribute to the literature on collective narcissism, which is similar to individual narcissism with respect to the belief in the exaggerated self-greatness and the desire for external recognition, but it extends such beliefs and desires to an ingroup level [23]. Blind patriotism is a special form of identity attachment, and it is closely related to collective narcissism [42]. While satisfying the fundamental psychological need for dignity, collective narcissism is associated with intensified sensitivity to group criticism and a stronger tendency towards motivated self-serving beliefs. Such patterns are manifested in the current identity politics and growing nationalisms around the world [10, 42]. Understanding Chinese nationalism as collective narcissism is helpful in comparing its common features with other identity politics, such as populist movements, racism and xenophobia at the global level. The underlying sociopsychological process is driven by the personal need for dignity and the desire for belonging, rather than a grand political agenda. It is not unique to Chinese nationalism and can therefore be expected to survive even without the support of the regime.

Identity attachment plays a crucial role in belief formation in the cognitive-motivational framework. As our study shows, both intergroup conspiracy beliefs and perceived objectivity are the most robust predictors of the overall rating of the book. These factors are in turn highly related to blind patriotism. Perceiving critics as liars and believing in conspiracy theories that favor the ingroup go hand in hand, as shown in our survey. Such conspiracy beliefs are not driven by a lack of cognitive ability, as most respondents in our sample are highly educated. This is consistent with the literature on motivated reasoning and beliefs, which argues that people choose what to believe in order to avoid cognitive dissonance, to maintain their identity, and to signify their loyalty to important ingroups [43, 44]. This is why the deeply-rooted “anti-China force” narrative found substantial resonance with our participants. More than 60% of our respondents were concerned about this, especially if they perceive Westerners as “happy to see China decaying” and as not understanding “the feelings of Chinese people.” This ‘zero-sum game’ thinking style which divides between ‘them’ and ‘us’ is not unique to China, and it is a potential source of conflict. International collaborations at institutional, professional, and societal levels are crucial to promote sustainable development and to cope with global crisis such as COVID-19 pandemic [45–53]. Fostering a common group identity at an international level can promote mutual trust and understanding, reduce biased beliefs, and improve effective collaborations [15, 54–56].

## Conflict of Interest

All authors of this paper do not have a conflict of interest to declare.

## Footnotes

1 The word “positive energy” (zheng neng liang) has been a popular catchphrase since 2012. Although this phrase was initially nonpolitical and grassroot in nature, the state, and in particular Xi Jinping’s regime, has actively used it to promote positive sentiment towards the state-party, and to disperse critiques, unhappiness and dissatisfaction as illegitimate “negative energy” (fu neng liang), reminiscent of a brave new world as depicted by Aldous Huxley. It is a remarkable example of how the state cleverly hijacked pop-culture and steered the public discourse to increase its own legitimacy [25]. Positive energy is explicitly used to promote patriotism, especially in cyberspace. A recent example is that Douyin (Chinese TikTok), a highly popular short-video platform, created a Positive Energy section [26]. How influential is this emerging cyber-nationalism? As such catchphrases are widespread in Chinese media, we expect that more exposure to Chinese media would increase the perception of “negative energy” from criticism, even as mild as that in the *Wuhan Diary*.

2 We compute the variable “perceived positive Western sentiment” as the difference between responses to the first and second statement.

3 We didn’t include the shame and blame questions because they are not significantly correlated to any variables in Table 4.
